# Toxicity Evaluation of Pig Slurry Using Luminescent Bacteria and Zebrafish

**DOI:** 10.3390/ijerph110706856

**Published:** 2014-07-03

**Authors:** Wenyan Chen, Qiang Cai, Yuan Zhao, Guojuan Zheng, Yuting Liang

**Affiliations:** 1 School of Environmental and Safety Engineering, Changzhou University, No. 1 GeHu Road, Wu Jin District, Changzhou 213164, Jiangsu, China; E-Mails: chen13812823711@163.com (W.C.); zhaoyuan12@tsinghua.org.cn (Y.Z.); 2 Yangtze Delta Region Institute of Tsinghua University, No. 705, Yatai Road, Nanhu District, Jiaxing 314006, Zhejiang, China; E-Mail: zhengguojuan.zj@gmail.com; 3 Water Science and Technology Lab, No. 705, Yatai Road, Nanhu District, Jiaxing 314006, Zhejiang, China; 4 Institute of Soil Science, Chinese Academy of Science, No. 71, Beijing East Road, Nanjing 210008, Jiangsu, China; E-Mail: liangyuting@tsinghua.org.cn

**Keywords:** pig slurry, luminescent bacteria, zebrafish, toxicity reduction

## Abstract

Biogas slurry has become a serious pollution problem and anaerobic digestion is widely applied to pig manure treatment for environmental protection and energy recovery. To evaluate environmental risk of the emission of biogas slurry, luminescent bacteria (*Vibrio fischeri*), larvae and embryos of zebrafish (*Danio rerio*) were used to detect the acute and development toxicity of digested and post-treated slurry. Then the ability of treatment process was evaluated. The results showed that digested slurry displayed strong toxicity to both zebrafish and luminescent bacteria, while the EC_50_ for luminescent bacteria and the LC_50_ for larvae were only 6.81% (*v/v*) and 1.95% (*v/v*) respectively, and embryonic development was inhibited at just 1% (*v/v*). Slurry still maintained a high level of toxicity although it had been treated by membrane bioreactor (MBR), while the LC_50_ of larvae was 75.23% (*v/v*) and there was a little effect on the development of embryos and *V*. *fischeri*; the results also revealed that the zebrafish larvae are more sensitive than embryos and luminescent bacteria to pig slurry. Finally, we also found the toxicity removal rate was higher than 90% after the treatment of MBR according to toxicity tests. In conclusion, further treatment should be used in pig slurry disposal or reused of final effluent.

## 1. Introduction

Due to limited fossil energy resources, renewable bio-energy technologies are regarded as alternative of fossil fuels in the future [1,2]. In recent years, biogas based on methane fermentation is becoming an attractive energy resource in many nations worldwide [3]. In China, there are more than 1.5 × 10^4^ biogas projects based on livestock and poultry breeding [4], which was expected to alleviate the energy shortage and reduce the emission of the waste.

However, it still poses environmental risk in biogas project even slurry was successfully anaerobically digested. Zhong *et al*. found there were high concentrations of heavy metals, such as Cd Cu, Ni, and Zn in the digested slurry [5]. In addition, some toxic organic pollutants also influence the effective utilization of biogas slurry, such as polychlorinated dibenzo-p-dioxins (PCDD), polychlorinated biphenyls (PCB), polyaromatic hydrocarbons (PAH), perfluorinated alkyl compounds (PFCs), linear alkylbenzene sulfonates (LASs), nonylphenols and nonylphenol ethoxylates (NP/NPEOs) and polybrominated diphenyl ethers (PBDEs) [6]. Recent research has focused on treatment methods for slurry, which could be potential requirement of the risk evaluation of slurry, especially toxicity.

On the other hand, some useful toxicity evaluation methods have been applied to the industrial effluents. Recent study finds the treated olive mill wastewater still had a certain degree of toxicity to *Vibrio fischeri* [7]. Similarly, it was possible that post-treated slurry would be still not suitable to release to river although it had been treated and reach discharge standard. Thus, biological toxicity tests *in vivo* and *in vitro* need to be applied to slurry management.

Bulich, A.A. *et al*. firstly reported the possibility that *Phototobacterium phosphoreum* could be applied to the toxicity evaluation of wastewater in the 1800s [8], more and more model organisms were developed. Up to now, more than eight methods have been developed and applied to different industrial effluents. Main bio-indicators could be luminescent bacteria [9], algae [10], *Daphnia magna* [11,12] and fish.

In these methods, the luminescent bacterium test, or Microtox test is one of the assays that frequently used for the acute toxicity assessment of environmental samples such as water and sediments [13], as well as pure compounds [14], for its relatively inexpensive, well-reproducible results, and fast testing procedure. The luminescence of bacteria depends on the existence of Adenosine triphosphate (ATP), fluorescein (FMN) and luciferase, so any factors that interfere or damage the respiration of the bacteria or physiological process can reduce the activity of luminescent bacteria. There are many different kinds of luminescent bacteria, such as *Vibrio*, *Photobacterium*, *Shewanella*, *Xetorhabdus*, and the most of *Vibrio*, *Photobacterium*, *Shewanella* were marine bacteria. So far, *Photobacterium phosphoreum*, *Vibrio fischeri* (ISO 11348-3) [15], and *Vibrio qinghaiensis* were widely used into total toxicity evaluation of different industrial effluent, such as textile finishing industry [16], tannery wastewaters [17] and so on.

For further evaluation of health risk, toxicity tests with early development stage of aquatic organisms have been introduced as a faster and more cost-effective way. Moreover, study showed that the early developmental stages of fish are often the most sensitive to toxic effects [18]. For relatively large, robust embryos and rapid embryonic development, zebrafish could be an ideal vertebrate model organism; moreover, transparent body is easily observed when zebrafish is developing outside their mother fish [19,20,21]. It was reported that some indexes, such as embryos production, atretic oocytes and altered ovarian histology and embryos mortality, could be useful to evaluate pharmaceutical mixture and municipal wastewater [22].

In this study, biological toxicity testing method was introduced to pig slurry, where related report on toxicity of slurry is still lack of study now. Here, *V*. *fischeri*, newly hatching larvae of zebrafish was used to evaluate acute toxicity of pig slurry, and embryos (1 hpf, 1 h post-fertilization) of zebrafish was for development toxicity. Using these methods, both digested and post-treated slurry were investigated to provide useful information about health risk of slurry and ability of the treatment process to reduce the toxicity of pig slurry, combined with physicochemical indexes (chemical oxygen demand (COD), ammonia nitrogen (NH_3_-N), total phosphorus (TP), *etc*.).

In this study, biological toxicity testing method was introduced to pig slurry, where related report on toxicity of slurry is still lack of study now. Here, *V*. *fischeri*, newly hatching larvae of zebrafish was used to evaluate acute toxicity of pig slurry, and embryos (1 hpf, 1 h post-fertilization) of zebrafish was for development toxicity. Using these methods, both digested and post-treated slurry were investigated to provide useful information about health risk of slurry and ability of the treatment process to reduce the toxicity of pig slurry, combined with physicochemical indexes (chemical oxygen demand (COD), ammonia nitrogen (NH_3_-N), total phosphorus (TP), *etc*.).

## 2. Experimental Section

### 2.1. Collection and Determination of Physicochemical Index of Samples

All samples of pig slurry were collected in Xinxin Forage Corporation in Jiaxing City of China. And before, pig slurry was pretreated by solid-liquid separation. Anaerobically digested and post-treated slurry (a 43 L membrane bio-reactor) were collected respectively. Digested slurry was collected in the outlet of anaerobic digester. Treated slurry was collected in the outlet of membrane bioreactor. Each index was determined three times respectively. After collection, physic-chemical variables were determined. Conductivity, pH, NH_3_-N, TP and COD were determined by conductivity meter (Monitoring and analysis method of water and waste water, in Chinese), glass electrode method (GB/T6920-1986, in Chinese), nessler’s reagent colorimetric method (GB/T7479-1987, in Chinese), ammonium molybdate spectrophotometric method (GBT11893-1989, in Chinese) and potassium dichromate method (GB/T11914-1989, in Chinese) respectively. And then the samples were stored at 4 °C until used. Before the toxicity experiments, all samples were diluted, where de-ion water was used in luminescence experiment, and standard dilution water [23] was used in zebrafish experiment.

### 2.2. Luminescent Bacteria

Freeze-dried marine luminescent bacteria (*V*. *fischeri* NRRL B-11177) were made in ampoule using freeze-drier (FD-5/8., Beijing Boyikang Test Co., Beijing, China). After the recovery of the freeze-dried powder, the initial luminous intensity needed to be between 2 × 10^6^ and 5 × 10^6^, which was detected by GloMax-Multi Detection System (Promega Co., Wisconsin, WI, USA) with a 96-well microplate (Corning/Costar Co., New York, NY, USA).

### 2.3. Zebrafish

Adult zebrafish (AB strain) were maintained in a recirculating aquaculture system (Aquaneering Co., San Diego, CA, USA). In incubation process, the 12 h light period was followed 12 h dark period per day. In the light period, the fish were fed with freshly hatched shrimp eggs and flake fish food (Tetra, Melle, Germany), twice and once respectively. The incubation temperature was controlled at 28 ± 0.5 °C.

### 2.4. Fertilized of Zebrafish Embryos

To hatch zebrafish embryos, one adult female fish and one adult male fish were placed in the same box. After the formation of zygote, embryos were washed 2–3 times by standard dilution water, for removing residues. Finally, normal developed fertilized eggs which were observed by the TS100-F microscope (Nikon, Tokyo, Japan) were collected for subsequent experiments.

### 2.5. Toxicity Tests

#### 2.5.1. Luminescent Bacteria Toxicity Test

Luminescent bacteria test was performed using 96-well microplate on the GloMax-Multi Detection System. Due to high toxicity of slurry and in order to eliminate the effect of the color and density on results, filtered samples were diluted to avoid complete inhibition. In this paper, volume percentage of sample in de-ion water was adopted to represent dilution degree, where raw sample is 100% *v/v* and de-ion water which was used as control sample was 0% *v/v*. The procedure in detail which was referring to research of Froehner *et al*. [24] was as follows: 100 μL de-ion water as blank controls was added to the first row of microplate, 100 μL sample with various dilution(respectively 100%, 50%, 25%, 12.5%, 6.25%, *v/v*) were added to the second, third, fourth, fifth and sixth row of microplate respectively. And then 100 μL bacteria suspension were added to each test well. After 15 min exposure, the luminescence intensity was measured. All of tests were repeated three times, while average luminescence intensity was adopted to dose-effect plot. Finally, the toxicity of slurry was characterized by relative luminous intensity and the concentration for 50% of maximal effect (EC_50_).

#### 2.5.2. Larvae of *D. Rerio* Acute Toxicity Test

The *D*. *rerio* 96 h acute toxicity test was carried out according to the procedure described in ISO7346-1 [25]. To detect toxicity using zebrafish larvae, digested and post-treated slurry were diluted to a series of exposure solutions to avoid complete inhibition due to high toxicity of slurry. Here volume percentage of sample in standard dilution water was adopted to represent dilution degree, where raw sample of digested and post-treated slurry is 100% *v/v* and standard dilution water which was used as control sample was 0% *v/v*. In exposure experiment, ten normally developed larvae were transferred to each culture dish (100 mm) containing 15 mL sample with different dilution degree. The number of dead larvae was counted at 24 h, 48 h, 72 h and 96 h after exposure to slurry. In this experiment, five different dilution degrees were introduced to evaluate the toxicity of slurry.

#### 2.5.3. Toxicity Test of Zebrafish Embryos

To detect toxicity using zebrafish embryos, diluted samples were needed to be prepared to avoid complete inhibition due to high toxicity of slurry. Normal embryos (at approximately 1 hpf) were kept in 24 well cell culture plates, with one embryo per well. Each well contained 1ml control or exposure wastewater. Two replicates for the controls and exposure groups were used. For each control and exposure group, the early embryonic development was observed by the TS100-F microscope and mortality was recorded at an interval of 24 h. After 72 h exposure experiments, mortality, hatching rate and malformation rate of embryos in each group were recorded. Similarly, five different dilution degrees were adopted [26].

### 2.6. Methods of Toxicity Evaluation

The toxicity of pig slurry on zebrafish larvae was evaluated using 96 h lethal concentration 50 (LC_50_), for *V*. *fischeri* and zebrafish embryos, EC_50_ and ELC_50_, HEC_50_ and MEC_50_ were used respectively. In this paper, ELC_50_, HEC_50_ and MEC_50_ were used to represent the concentration of wastewater for 50% of embryonic mortality, hatching and malformation respectively while exposed to the pig slurry. Finally, toxicity unit (TU) was used to represent the toxicity directly. TU was calculated according to the formula as follows [27]:

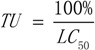
(1)

If the inhibition of luminescence intensity for *V*. *fischeri*, hatching rate for zebrafish embryos, mortality for zebrafish larvae were lower than 50% and malformation rate for zebrafish embryos was lower than 50% exposed to pig slurry, it showed LC_50_ (EC_50_) couldn’t be calculated, TU was calculated according to the formula as in Equation (2) [27]:

*TU* = *RE* × 100 × 0.02 (2)

where RE was the relative inhibition rate of *V*. *fischeri* luminosity and death rate of larvae and embryos of zebrafish (%)

And the toxicity remove rate was calculated according to the formula as follows:

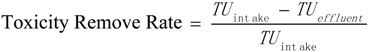
(3)

### 2.7. Statistical Analysis

Using origin 8.0 (Origin Lab, Northampton, MA, USA), the median lethal concentration (LC_50_) and the median effect concentration (EC_50_) on *D*. *rerio* and luminescent bacteria of pig slurry were calculated, followed with one-way ANOVA in SPSS 16.0. Here statistical significance difference of exposure group to the control group was set to *p <* 0.01.

## 3. Results

### 3.1. Physicochemical Characterization of Pig Slurry Effluents

The calculated values of physicochemical indexes were listed in Table 1. It showed that pollutants in the pig slurry were reduced mostly after the treatment of MBR, where the value of NH_3_-N and COD of raw water were reduced 94.5% and 86.0% respectively. On the other hand, the allowed values of NH_3_-N and COD are 80 mg/L and 400 mg/L respectively, according to discharge standard of water pollutants for livestock and poultry breeding(GB 18596-2001) [28]. It meant the effluent could be discharged legally.

**Table 1:** Routine physicochemical index of test slurry.

	pH	Conductivity (ms∙cm^−1^)	NH_3_-N (mg∙L^−1^)	TP (mg∙L^−1^)	COD (mg∙L^−1^)
Digested slurry	7.69	10.01	845	152.45	2667
Post-treated slurry	7.27	5.97	46.2	24.04	373

### 3.2. Effect on Luminescent Bacterial Exposed to Pig Slurry

**Figure 1:**
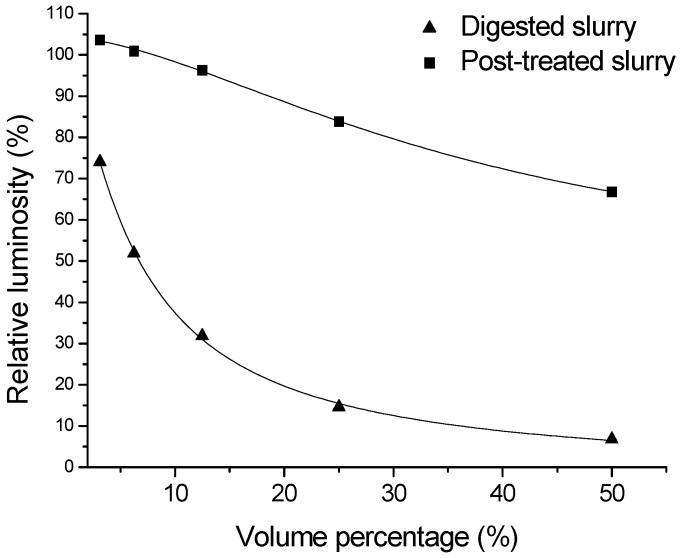
Plot of relative luminosity *vs*. volume percentage of digested and post-treated slurry where the curves were fitted to Logistic function. Results are means standard deviations (*n =* 3).

The dose-response curves after 15 min exposure were plotted using logistic model, shown in Table 2 and Figure 1. Dose-response curve is nonlinear fitted by Logistic regression model (Equation (4)):

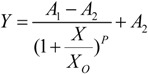
(4)

The relative luminosity of *V*. *fischeri* exposed to digested and post-treated slurry, were 6.9% and 66.8% respectively, which means both of them had a certain degree of inhibitory effect on the *V*. *fischeri*. Table 2 showed good statistical significance for regression model while the R^2^ was higher than 0.99. And the toxicity could be divided into the following four categories based on EC_50_ [7]: (i) high toxicity for samples with an EC_50_ < 25%; (ii) moderate toxicity, EC_50_ 25% to 75%; (iii) low toxicity with a 75% to 100% of EC_50_; (iiii) non-toxicity, EC_50_ can’t be calculated. The EC_50_ of digested slurry to *V*. *fischeri* was 6.81% dilution, which means high toxicity. In contrast, relative luminosity was inhibited only 40.09% by raw sample for post-treated slurry, which is lower than 50%. So EC_50_ of post-treated slurry can’t be calculated, which meant non-toxicity to luminescent bacteria. According to the TUs, toxicity remove rate of was 94.6% for MBR-treated slurry. Compared with the physicochemical characteristics of slurry, the similar remove effect was proved.

**Table 2:** Nonlinear dose-response models with some statistics, EC_50_ and TU of test wastewater to luminescent bacteria.

	A_1_	A_2_	X_0_	*p*	R^2^	EC_50_ (%)	TU ^a^
Digested slurry	108.22	−1.79	6.09	1.19	99.76%	6.81 ± 1.56	14.68
Post-treated slurry	104.30	43.02	38.04	1.66	99.83%	No inhibition	0.80

^a^ In this table, TU means the toxic unit of digested and post-treated slurry to test organism.

### 3.3. Effect on Zebrafish Larvae Exposed to Pig Slurry

**Figure 2:**
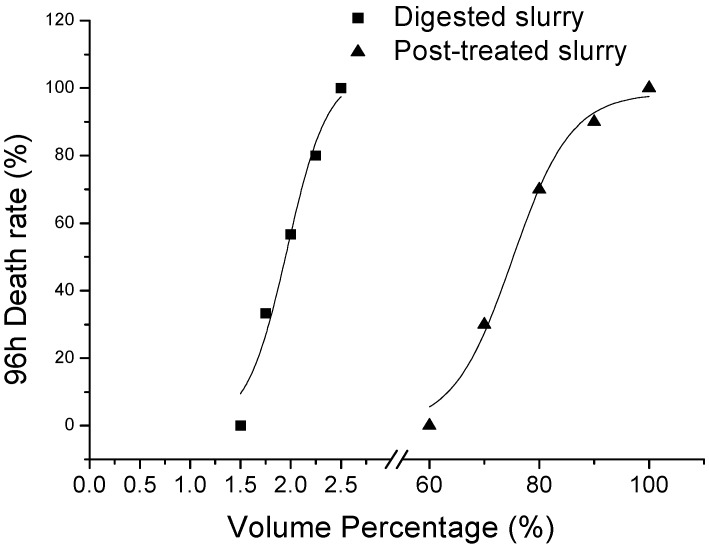
Plot of 96h-death rate *vs*. volume percentage of digested and post-treated slurry where the curves were fitted to Logistic function.

The results of 96 h acute toxicity test of zebrafish larvae were showed in Figure 2 and Table 3. As Figure 2 described, when larvae were exposed to digested slurry (2.5%, *v/v*), the mortality had already reach 100%. On the other hand, the same effect on mortality could be found while the volume percentage was up to 100% for post-treated slurry. Table 3 showed LC_50_ of larvae exposed to digested and post-treated slurry were different, 1.95% and 75.23% respectively. According to TU, treatment of MBR reduces the toxicity by 97.5%. The results showed the high degree of reduction of toxicity after treatment by MBR. It showed that the reduction rate calculated by 96h acute toxicity test of larvae was not much different from by luminescent bacterial acute toxicity test and physicochemical indexes.

**Table 3:** Effect on larvae exposed to digested and post-treated slurry.

	LC_50_ (%)	TU
Digested slurry	1.95	51.33
Post-treated slurry	75.23	1.33

### 3.4. Effect on the Early Development of Embryos Exposed to Pig Slurry

Post-treated slurry showed a certain degree of toxicity to mortality, hatching and malformation (45%, 25% and 41.7% respectively in Table 4) compared with control group. In 72 h experiments with embryos exposed to digested and post-treated slurry, there was a significant effect on embryonic mortality, hatching rate and malformation rate, while the ELC_50_, HEC_50_ and MEC_50_ of embryos exposed to digested slurry were 3.48%, 1.32% and 3.47% respectively. And post-treated slurry showed no inhibition to the embryos of ELC_50_ and MEC_50_. However, the EC_50_ of hatching rate of embryos exposed to post-treated slurry reached 31.81% (Table 5).

**Table 4:** Toxicity of digested and post-treated slurry to zebrafish embryo.

	Volume Fraction (%)	Embryonic Mortality (%)	Hatching Rate (%)	Malformation Rate (%)
Control	0	0	100	0
Digested slurry	1.0	5	55	10.5
	2.0	20	35	18.8
	3.0	40	20	41.7
	4.0	60	0	62.5
	5.0	75	0	100
Post-treated slurry	6.25	5	80	0
	12.5	10	70	5.6
	25.0	20	55	6.3
	50.0	35	40	23.1
	100.0	45	25	41.7

**Table 5:** Effects of digested and post-treated slurry on mortality, hatching and malformation rate of zebrafish embryo.

	ELC_50_ (%) ^a^	HEC_50_ (%) ^b^	MEC_50_ (%) ^c^
Digested slurry	3.48	1.32	3.47
Post-treated slurry	No inhibition	31.81	No inhibition

Notes: ^a^ ELC_50_ represents the concentration of wastewater for 50% of embryonic mortality; ^b^ HEC_50_ represents the concentration of wastewater for 50% of hatching; ^c^ MEC_50_ represents the concentration of wastewater for 50% of malformation.

**Figure 3:**
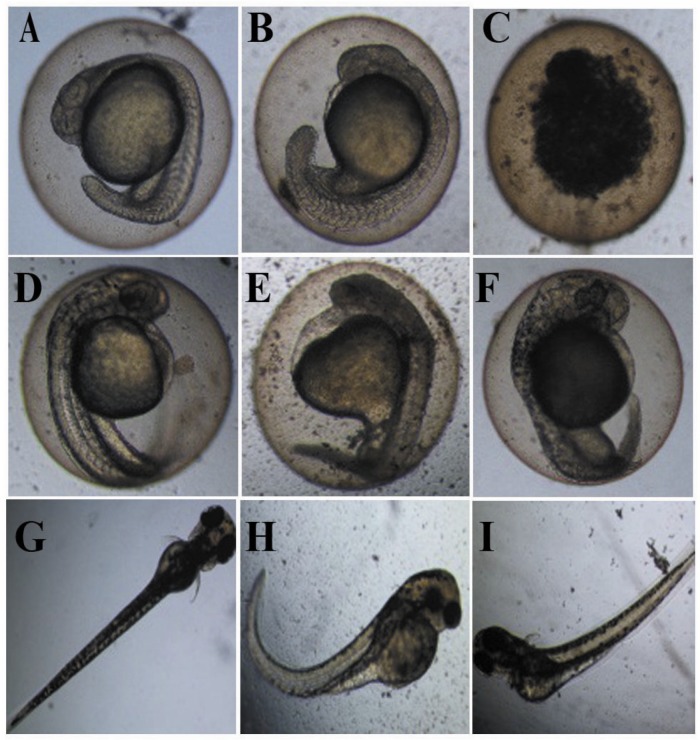
Pictures of the early embryonic development of zebrafish embryo exposed to post-treated slurry ((**a**) 24 h-normal development; (**b**) 24 h-no extension of the embryonic tail; (**c**) 24 h-coagulated egg; (**d)** 48 h-normal development;(**e**) 48 h-pericardial edema; (**f**) 48 h-pigment deposition; (**g)** 72 h-normal development; (**h**,**i**) 72 h-malformed larvae treated with wastewater)).

According to the TU in Table 6, the remove rate of toxicity to mortality, hatching and malformation were 96.9%, 95.9% and 97.1% respectively. With microscopic observation, malformed embryos displayed the different status, such as the 24 h no extension of the embryonic tail, 24 h coagulated egg, 48 h pericardial edema, 48 h pigment deposition, 72 h-malformed larvae. On the other hand, 24 h development of eyespot and 48 h otoconial development were not found in this experiment (Figure 3).

**Table 6:** TUs of digested and post-treated slurry to mortality, hatching and malformation rate of zebrafish embryo.

	TU		
	Mortality	Hatching	Malformation
Digested slurry	28.74	75.76	28.82
Post-treated slurry	0.90	3.14	0.83

## 4. Discussion

The application of biogas engineering has received considerable attention in recent years, while there are many controversies about the advantages and disadvantages of biogas slurry. In some studies, slurry could be used to give rise of high yields to crops.J. Abubaker *et al*. [29] conducted a study on the fertilizing performance of pig slurry and mineral fertilizer in terms of spring wheat yield, in conclusion, pig slurry gave the overall highest yields to wheat. Gobernaa *et al*. [30] compared biogas digestates with fresh manure to the inhibition of pathogenic bacteria in soils, and found anaerobic digestion significantly sanitized the manure by completely eliminating cultivable *E*. *coli* and *Salmonella*. However, toxic pollutants in the biogas slurry could be dangerous to environment. Many different pollutants had been found in slurry. For evaluation of availability of slurry, some researches were conducted. Using *Daphnia magna*, A.I. De la Torre *et al*. [31] stated that toxicities of pig slurry were higher than urban effluents and lower than industrial effluents.

For evaluation of acute and development toxicity of slurry, *V*. *fischeri*, zebrafish larvae and embryos were exposed to digested and post-treated slurry respectively. In this study, different trophic level organisms showed different sensitivity to digested and post-treated slurry. The sensitivity order of the three test organisms was larva > embryo > *V*. *fischeri*. The difference of larva and embryo of zebrafish could result from membrane out of the embryo, which selectively restrict big molecule compounds to enter into embryo, which could be regarded as protective effect on the embryo [32], as had been reported by Wiegand C *et al*. [33,34]. There is little literature on the conductivity requirements for zebraﬁsh larva and embryo, but adult zebrafish are tolerant to conductivity ranging from 400 μS∙cm^−1^ to more than 1000 μS∙cm^−1^ [35]. In this study, conductivity of digested and post-treated slurry was both far higher than 1000 μS∙cm^−1^. In addition, there was a correlation between conductivity and salinity generally. And *V*. *fischeri was regarded as* marine bacteria, so probably the higher sensitivity of fish versue *V*. *fischeri* could be mainly due to the different salinity tolerance. It showed the use of the marine bacteria tests is very important because it can avoid effect of salinity on test organism, this conclusion has also been confirmed by Pardo *et al*. [36].

**Figure 4:**
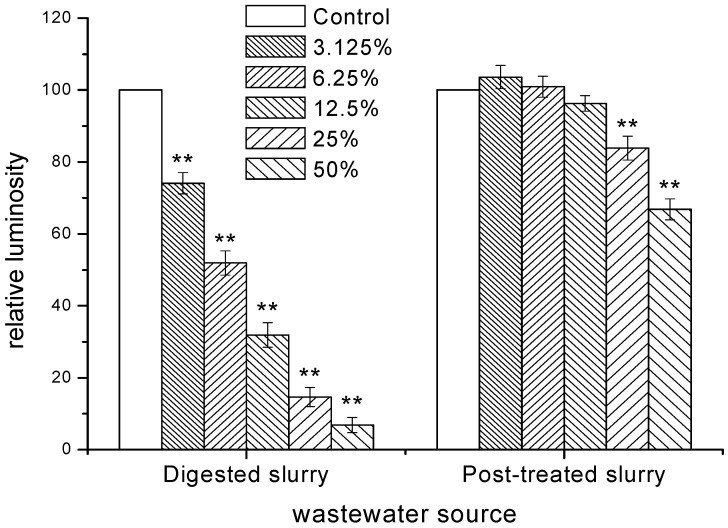
Variance analysis for relative luminosity of *V*. *fischeri* exposed to digested and post-treated slurry. Data are mean standard deviation; *n =* 3, per group. ** *p <* 0.01, others were no significant differences.

Result also demonstrated that pig slurry had a high toxicity to both *V*. *fischeri* and zebrafish. The toxicity of digested slurry to *V*. *fischeri* was 14.68 TUs, which was higher than the TU of phenol (12.5 TUs) and lower than that of dimethyldiuron (16.23 TUs). Meanwhile, the toxicity of post-treated slurry to *V*. *fischeri* was 0.80 TU, which was between the TU of polyethylene glycol (0.78 TU) and cypermethrin (0.91 TU) [37]. In addition, relative luminosity of *V*. *fischeri* exposed to post-treated slurry, was very significantly inhibited comparing with the de-ion water. On the other hand, relative luminosity of *V*. *fischeri* was higher than 100% (ANOVA, *p <* 0.01; Figure 4), when effluent was diluted to a certain degree of volume percentage. Some nutrients existed in pig slurry could enhance the cell activity of luminescent bacteria. Similar results could be observed in pure compounds experiments. For example, relative luminosity of *V*. *fischeri* was higher than 100% when the concentration of phenol was lower than 0.005 mg/L [38]. Probably because that the inhibition on biological effect was lower, and bacterial recovery effect is stronger.

In addition, zebrafish larvae were all dead when exposed to digested and post-treated slurry, the same effect as the zebrafish embryos exposed to digested slurry, however the mortality of embryos exposed to post-treated slurry was lower than 50%. Meanwhile, effects of pig slurry on the early developing zebrafish embryos were observed. The experiment of embryos exposed to post-treated slurry also showed that the hatching rate was lower than 30% and malformation rate was more than 40%. According to reported development toxicity test of urban sewages using zebrafish embryos, hatching rate of embryos was more than 80% and malformation rate of embryos was lower than 20% respectively [39]. It showed the toxicity of pig slurry was higher than urban sewages.

Moreover, 96 h LC_50_ and toxicity of post-treated slurry to the zebrafish larvae were higher than 70% and lower than 2 TUs respectively. According to acute toxicity test of zebrafish larvae exposed to fat-plant effluent, showed that the value of LC_50_ of larvae exposed to effluent was lower than 70%, reported by Şişman T. *et al*. [40]. In addition, adult zebrafish were used to evaluate the toxicity of industry effluent, such as electronic and electroplate effluent, the result showed the TU of effluent was 2.54 [41]. Then considering that zebrafish larva is more sensitive than adult zebrafish. Thus, toxicity of post-treated slurry was lower than industrial effluents. And it showed that it was necessary to apply treatment to slurry, owing to high environmental risk.

Apparently, we could find the toxicity of digested slurry was reduced mostly after the treatment of MBR according to the luminescent bacteria toxicity test and zebrafish larvae acute toxicity test, the removal rate was 94.6% and 97.5% respectively, meanwhile according to the results of physicochemical index determination, post-treated slurry could be discharged legally. However, mortality of larvae still reached 100% when exposed to post-treated slurry. Besides other toxics, such as ammonia could be responsible for the toxicity of pig slurry. Recent study shows that ammonia can be physiologically harmful to *Hypophthalmichthys molitrix* larvae, through increasing the concentration of reactive oxygen species and oxidative damage products such as lipid peroxides [42]. Among the physiochemical characteristics of the slurry (NH_3_-N, TP and COD), there was a high correlation between the toxicity removal rate and reduction rate of NH_3_-N. It meant the decrease of concentration of NH_3_-N, toxicity of pig slurry has also been reduced. So result that NH_3_-N made much greater contribution on slurry toxicity could be obtained and further treatment should be used in pig slurry disposal or reused of final effluent.

In this paper, risk information was acquired while evaluation method was applied to pig slurry. However, more organisms for quantitative assessment are necessary. So a battery of bioassays based on organisms belong to different trophic levels, are strongly suggested, which could get a risk score by the application of a synthetic index for toxicity [43], combining algae, *Daphnia magna* and so on.

## 5. Conclusions

Pig slurry had showed different toxicity effect on *V*. *fischeri* and zebrafish. And larva was the more sensitive one to all the test wastewater, followed by embryo of zebrafish and finally, *V*. *fischeri*. Furthermore, both *V*. *fischeri* and zebrafish could be used in the toxicity reduction and risk assessment of pig slurry, and provided suggestions for its treatment process. Based these methods, risk information of slurry could be acquired. Here digested slurry displayed strong toxicity to luminescent bacteria, zebrafish larva and embryo. On the other hand, post-treated slurry still showed low toxicity although it has reached the discharged standard, and toxicity remove rate has reached up more than 90%. Considering that, too much discharged slurry could exceed rural river carrying capacity, advanced treatment for slurry, such as ion exchange technique, wet oxidation process, biological nitrogen removal system and so on, are suggested.

## References

[1] Pachauri R.K., Reisinger A. Contribution of Working Group III to the Fourth Assessment Report of the Intergovernmental Panel on Climate Change: Mitigation of Climate Change. Proceedings of the Intergovernmental Panel on Climate Change.

[2] Mathiesen B.V., Lund H., Karlsson K. (2011). 100% Renewable energy systems, climate mitigation and economic growth. Appl. Energy.

[3] Chandra R., Vijay V., Subbarao P., Khura T. (2011). Performance evaluation of a constant speed IC engine on CNG, methane enriched biogas and biogas. Appl. Energy.

[4] Zhang G.Z., Wu S.B., Wang H.L., Wei S.Q., Wang K.Y., Long Y., Deng L.W. (2009). Survey and analysis on slate quo of public intention for utilizing digestate from large and medium size biogas plant. China Biogas.

[5] Nkemka V.N., Murto M. (2010). Evaluation of biogas production from seaweed in batch tests and in UASB reactors combined with the removal of heavy metals. J. Environ. Manag..

[6] Suominen K., Verta M., Marttinen S. (2014). Hazardous organic compounds in biogas plant end products—Soil burden and risk to food safety. Sci. Total Environ..

[7] Mekki A., Dhouib A., Feki F., Sayadi S. (2008). Assessment of toxicity of the untreated and treated olive mill wastewaters and soil irrigated by using microbiotests. Ecotoxicol. Environ. Saf..

[8] Bulich A.A., Isenberg D. (1981). Use of the luminescent bacterial system for the rapid assessment of aquatic toxicity. ISA Trans.

[9] Tigini V., Giansanti P., Mangiavillano A., Pannocchia A., Varese G.C. (2011). Evaluation of toxicity, genotoxicity and environmental risk of simulated textile and tannery wastewaters with a battery of biotests. Ecotoxicol. Environ. Saf..

[10] Meric S., de Nicola E., Iaccarino M., Gallo M., di Gennaro A., Morrone G., Warnau M., Belgiorno V., Pagano G. (2005). Toxicity of leather tanning wastewater effluents in sea urchin early development and in marine microalgae. Chemosphere.

[11] Oral R., Meriç S., de Nicola E., Petruzzelli D., Della Rocca C., Pagano G. (2007). Multi-species toxicity evaluation of a chromium-based leather tannery wastewater. Desalination.

[12] Ra J.S., Kim S.D., Chang N.I., An K.G. (2007). Ecological health assessments based on whole effluent toxicity tests and the index of biological integrity in temperate streams influenced by wastewater treatment plant effluents. Environ. Toxicol. Chem..

[13] Dutka B., Kwan K., Rao S., Jurkovic A., McInnis R., Palmateer G., Hawkins B. (1991). Use of bioassays to evaluate river water and sediment quality. Environ. Toxicol. Water Qual..

[14] Kaiser K.L., Palabrica V.S. (1991). *Photobacterium phosphoreum* toxicity data index. Water Qual. Res. J. Can..

[15] Organisation for Economic Co-operation and Development (ISO) (2007). Water Quality-Determination of the Inhibitory Effect of Water Samples on the Light Emission of Vibrio fischeri (Luminescent Bacteria Test)—Part 3: Method Using Freeze-Dried Bacteria.

[16] Wang C., Yediler A., Lienert D., Wang Z., Kettrup A. (2002). Toxicity evaluation of reactive dyestuffs, auxiliaries and selected effluents in textile finishing industry to luminescent bacteria (*Vibrio fischeri*). Chemosphere.

[17] Arias-Barreiro C., Nishizaki H., Okubo K., Aoyama I., Mori I. (2010). Ecotoxicological characterization of tannery wastewater in Dhaka, Bangladesh. J. Environ. Biol..

[18] McKim J.M., Rand G.M. (1995). Early Life Stage Toxicity Tests. Fundamentals of Aquatic Toxicology: Effects, Environmental Fate and Risk Assessment.

[19] Chio C.P., Chen W.Y., Chou W.C., Hsieh N.H., Ling M.P., Liao C.M. (2012). Assessing the potential risks to zebrafish posed by environmentally relevant copper and silver nanoparticles. Sci. Total Environ..

[20] Kais B., Schneider K., Keiter S., Henn K., Ackermann C., Braunbeck T. (2013). DMSO modifies the permeability of the zebrafish (*Danio rerio*) chorion-implications for the fish embryo test (FET). Aquat. Toxicol..

[21] Selderslaghs I.W., Blust R., Witters H.E. (2012). Feasibility study of the zebrafish assay as an alternative method to screen for developmental toxicity and embryotoxicity using a training set of 27 compounds. Reprod. Toxicol..

[22] Galus M., Jeyaranjaan J., Smith E., Li H., Metcalfe C., Wilson J.Y. (2013). Chronic effects of exposure to a pharmaceutical mixture and municipal wastewater in zebrafish. Aquat. Toxicol..

[23] Gellert G., Heinrichsdorff J. (2001). Effect of age on the susceptibility of zebrafish eggs to industrial wastewater. Water. Res..

[24] Froehner K., Meyer W., Grimme L.H. (2002). Time-dependent toxicity in the long-term inhibition assay with *Vibrio fischeri*. Chemosphere..

[25] Organisation for Economic Co-operation and Development (ISO) (1996). Water Quality—Determination of the Acute Lethal Toxicity of Substances to a Freshwater Fish [Brachydanio rerio Hamilton-Buchanan (Teleostei, Cyprinidae)]—Part 1: Static Method.

[26] Weigt S., Huebler N., Braunbeck T., Landenberg F.V. (2010). Zebrafish teratogenicity test with metabolic activation (mDarT): Effects of phase I activation of acetaminophen on zebrafish *Danio rerio* embryos. Toxicology.

[27] Gao L., Li Z.L., Chen H.H., Chen H., Cha J.M., Wang Z.J. (2011). Toxicities of dye effluent on Japanese Medaka (*Oryzias latipes*) embryo and larava. Asian J. Ecotoxicol..

[28] Discharge Standard of Pollutants for Livestock And Poultry Breeding. http://english.mep.gov.cn/standards_reports/standards/water_environment/Discharge_standard/200710/t20071024_111807.htm.

[29] Abubaker J., Risberg K., Pell M. (2012). Biogas residues as fertilizers-Effects on wheat growth and soil microbial activities. Appl. Energy.

[30] Goberna M., Podmirseg S.M., Waldhuber S., Knappa B.A., Garcíab C., Insama H. (2011). Pathogenic bacteria and mineral N in soils following the land spreading of biogas digestates and fresh manure. Appl. Soil Ecol..

[31] De la Torre A., Jiménez J., Carballo M., Fernandez C., Roset J., Munoz M. (2000). Ecotoxicological evaluation of pig slurry. Chemosphere.

[32] Henn K., Braunbeck T. (2011). Dechorionation as a tool to improve the fish embryo toxicity test (FET) with the zebrafish (*Danio rerio*). Comp. Biochem. Phys. C: Toxicol. Pharmacol..

[33] Wiegand C., Pflugmacher S., Oberemm A., Steinberg C. (2000). Activity development of selected detoxication enzymes during the ontogenesis of the zebrafish (*Danio rerio*). Int. Rev. Hydrobiol..

[34] Wiegand C., Pflugmacher S., Giese M., Frank H., Steinberg C. (2000). Uptake, toxicity, and effects on detoxication enzymes of atrazine and trifluoroacetate in embryos of zebrafish. Ecotox. Environ. Saf..

[35] Richards F.M., Alderton W.K., Kimber G.M., Liu Z., Strang I., Redfern W.S., Winter M.J., Hutchinson T.H. (2008). Validation of the use of zebrafish larvae in visual safety assessment. J. Pharmacol. Toxicol. Methods..

[36] Pardo T., Clemente R., Alvarenga P., Bernal M.P. (2014). Efficiency of soil organic and inorganic amendments on the remediation of a contaminated mine soil: II. Biological and ecotoxicological evaluation. Chemosphere.

[37] Farré M., Barceló D. (2003). Toxicity testing of wastewater and sewage sludge by biosensors, bioassays and chemical analysis. Trac.-Trend. Anal. Chem..

[38] Zhang J.Y., Ding T.D., Liang L.Y., Wang F.P., Chen J. (2012). Response of aquatic ecosystem to phenol pollution at different concentration levels. Environ. Chem..

[39] Liu Z.P., Zhang S.L., Wu E.S., Yang J.H., Tang R. (2010). Toxicity study on urban sewage to larva of zebrafish. Environ. Sci. Tech..

[40] Şişman T., İncekara Ü., Yıldız Y.Ş. (2008). Determination of acute and early life stage toxicity of fat-plant effluent using zebrafish (*Danio rerio*). Environ. Toxicol..

[41] Fang Y.X., Ying G.G., Zhang L.J., Zhao J.L., Su H.C., Yang B., Liu S. (2012). Use of TIE techniques to characterize industrial effluents in the Pearl River Delta region. Ecotoxicol. Environ. Saf..

[42] Sun H.J., Yang W., Chen Y.F., Yang Z. (2011). Effect of purified microcystin on oxidative stress of silver carp *Hypophthalmichthys molitrix* larvae under different ammonia concentrations. Biochem. Syst. Ecol..

[43] Carballeira C., de Orte M., Viana I., Carballeira A. (2012). Implementation of a minimal set of biological tests to assess the ecotoxic effects of effluents from land-based marine fish farms. Ecotoxicol. Environ. Saf..

